# Insights into the Pathogenesis of Neurodegenerative Diseases: Focus on Mitochondrial Dysfunction and Oxidative Stress

**DOI:** 10.3390/ijms222111847

**Published:** 2021-10-31

**Authors:** Anamaria Jurcau

**Affiliations:** 1 Department of Psycho-Neurosciences and Rehabilitation, Faculty of Medicine and Pharmacy, University of Oradea, 410073 Oradea, Romania; anamaria.jurcau@gmail.com; 2 Neurology Ward, Clinical Municipal Hospital “dr. G. Curteanu” Oradea, 410154 Oradea, Romania

**Keywords:** mitochondrial dysfunction, oxidative stress, antioxidants, Alzheimer’s disease, Parkinson’s disease, amyotrophic lateral sclerosis

## Abstract

As the population ages, the incidence of neurodegenerative diseases is increasing. Due to intensive research, important steps in the elucidation of pathogenetic cascades have been made and significantly implicated mitochondrial dysfunction and oxidative stress. However, the available treatment in Alzheimer’s disease, Parkinson’s disease, and amyotrophic lateral sclerosis is mainly symptomatic, providing minor benefits and, at most, slowing down the progression of the disease. Although in preclinical setting, drugs targeting mitochondrial dysfunction and oxidative stress yielded encouraging results, clinical trials failed or had inconclusive results. It is likely that by the time of clinical diagnosis, the pathogenetic cascades are full-blown and significant numbers of neurons have already degenerated, making it impossible for mitochondria-targeted or antioxidant molecules to stop or reverse the process. Until further research will provide more efficient molecules, a healthy lifestyle, with plenty of dietary antioxidants and avoidance of exogenous oxidants may postpone the onset of neurodegeneration, while familial cases may benefit from genetic testing and aggressive therapy started in the preclinical stage.

## 1. Introduction

Aging associates a series of physiologic deficits and a variable degree of cognitive impairment, being also a major risk factor for neurodegenerative diseases such as Alzheimer’s disease (AD), Parkinson’s disease (PD), or amyotrophic lateral sclerosis (ALS) [1]. From the enormous amount of research aimed at unraveling the mechanisms of aging and neurodegeneration several pieces of the puzzle emerged, but we have still a long way to go to grasp the whole picture. However, it appears that oxidative damage induced by free radicals and mitochondrial dysfunction play a major role in both processes.

## 2. Normal Aging

Aging of the brain occurs at molecular, cellular, and histological levels [2]. It associates lower levels of neuronal metabolic activity, subtle alterations in neuronal structure in several neuronal circuits, as well as synaptic atrophy, cytoskeletal abnormalities, accumulation of fluorescent pigments, and reactive astrocytes and microglia [3,4]. Research points toward the hypothalamus as initiating and controlling the gradual decline of energy metabolism of the entire body [5,6]. Through the secretion of neurohormones, the connections with the endocrine system, and projections of the orexinergic nucleus to the reticular activating system [5], the hypothalamus regulates the stress levels, metabolism, sleep, and influences the subjectively perceived quality of life and establishing of social relationships [7,8,9]. Degeneration of the suprachiasmatic nucleus may additionally contribute to circadian rhythm disorders and impaired sleep [10,11,12]. Limiting sleep may cause neuronal toxic waste products to accumulate and limit neurogenesis in the aging brain, igniting a vicious cycle which augments the neurodegenerative process [13].

Additional factors contribute to the declining metabolism of the brain cells. Cerebral metabolism relies mainly on a constant supply of glucose and oxygen through the blood flow, and, to a limited extent, on lactate [14]. Neuronal glucose uptake is mediated by glucose transporters (GLUTs), after which it is converted into glucose-6-phosphate (G6P) by hexokinase. As such, the availability of ATP, depending on oxygen supply and mitochondrial oxidative phosphorylation (OXPHOS), interferes with glucose uptake [5,15]. OXPHOS produces considerable higher amounts of ATP as compared to glycolysis [16,17]. In aging, reduced metabolism can be caused by mitochondrial dysfunction and reduced ATP synthesis, as well as by vascular changes which lead to limited oxygen supply.

The high cellular energy expenditure in the nervous system, used for synaptic transmission (around 80% of the energy consumption of the brain) [18,19], synaptogenesis, and synaptic pruning [18,20] associates a high rate of generation of reactive oxygen species mainly by electrons leaked from the mitochondrial electron transport chain (ETC). The excessive generation of free radicals cannot be neutralized by the antioxidant defenses and leads to oxidative stress, implicated in aging since the 1950s, when Harman suggested that free radical-induced damage of biomolecules, such as proteins, lipids, and DNA, causes a reduction of their biochemical and physiological function in aging [21]. Indeed, research has demonstrated altered composition of phospholipids in the brains of aged humans and animals together with increased malondialdehyde (a marker of lipid peroxidation) generation, which forms deposits connected with intraneuronal lipofuscin [22], and elevated carbonyl residues (a marker of protein oxidation) [23]. In addition, aging decreases the antioxidant defense systems, such as the astrocytic glutathione system [24], which further potentiates oxidative stress [2].

Mitochondria undergo a series of age-related changes such as fragmentation or enlargement [25], increased mitochondrial DNA (mtDNA) oxidative damage [26], exhibit dysfunctions of the respiratory chain [27] and of calcium homeostasis [28]. These changes associate a reduction of the intracellular NAD^+^ levels which impairs the function of NA dependent enzymes such as sirtuins (SIRT) and histone deacetylases [29,30]. Sirtuins are highly evolutionary conserved enzymes involved in the regulation of lifespan and aging in different organisms, from yeast to mammals [31]. In mammals, SIRT 3, 4, and 5 are located in the mitochondria, SIRT 2 in the cytosol, and SIRT 1, 6, and 7 are located in the nucleus [32]. Research has shown that SIRT 1, mainly in the hypothalamus, is a key player in controlling aging and longevity in mammalian organisms [33,34].

Shortening of the telomeres, which promote chromosomal stability during cell replication [35], was initially disregarded as having important influence in the brain since neurons are essentially postmitotic cells which no longer replicate. However, this view has been challenged by demonstrating cell cycle activity in 10–20% of neurons in the cortex of healthy aging brains and in AD [36,37], as well as by the presence of neural stem cells in the subventricular and subgranular zones, in the choroid plexuses and meninges [38]. In addition, glial cells (especially microglia) do replicate actively, with telomeres gradually shortening after each cellular replication [39].

Aging induces also an inflammatory phenotype during which, in response to mutations and DNA damage, nuclear factor-κB (NF-κB) initiates the transcription of tumor necrosis factor-α (TNF-α) and various inflammatory interleukins (IL-1β, IL-6, IL-8) [40]. ROS have a crucial role in this process, since they phosphorylate and degrade IκB, which binds to and inactivates NF-κB [41]. Low amounts of ROS initiate pro-survival signaling cascades, the activated NF-κB suppressing c-Jun N-terminal kinases (JNKs) and apoptosis and upregulating antioxidant and anti-apoptotic genes such as manganese superoxide dismutase (MnSOD) [42]. However, high ROS concentrations activate NF-κB through protein kinases and initiate cellular stress signaling pathways. Damaged neurons alter the ionic balance in the interstitial space and lead to cytokine release, thereby activating microglia [43,44]. Although TNF-α has important roles in learning and synaptic plasticity [45], excessive microglial activation will cause degeneration of synapses and functional impairments, characteristic of aging and neurodegeneration [46]. In addition, the inflammatory responses induce other transcription factors, such as signal transducer and activator of transcription (STAT-1) and peroxisome proliferator-activated receptor-gamma (PPARγ), the latter playing an important role in mitochondrial biogenesis [47]. As such, the secretory phenotype associated with senescence (SASP), particularly in astrocytes, can trigger several age-related neurodegenerative diseases [48].

## 3. Mitochondria in the Brain

The high cerebral metabolic activity relies mainly on oxidative phosphorylation for ATP production. However, mitochondria exert other important functions in the brain as well. The constant signaling leads to continuous variations in the cytosolic calcium concentrations and mitochondria in collaboration with the endoplasmic reticulum have a crucial role in regulating neurotransmission by buffering calcium in presynaptic terminals and regulating the somato-dendritic calcium levels [49,50]. Moreover, mitochondria regulate cell cycle and control cell death [51]. In order to accomplish these diverse functions, maintenance of mitochondrial fitness is crucial, requiring efficient quality control mechanisms [52], achieved through mitochondrial biogenesis and fission (separation of a single organelle into two or more daughter organelles, essential for populating dividing or growing cells with an adequate number of mitochondria), fusion (a process through which mitochondria share essential components), and mitophagy (elimination of damaged mitochondria before they lead to apoptosis of the whole cell) [53,54]. In addition, mitochondria must be trafficked along axons to provide energy all across the axon [2].

### 3.1. Mitochondrial Respiratory Chain and ROS Production

A mitochondrion has a spongy outer mitochondrial membrane (OMM), which allows free movement of small ions and uncharged molecules, and an impermeable inner mitochondrial membrane (IMM), which envelops the mitochondrial matrix. Between the IMM and OMM lies the intermembrane space [55]. The mitochondrial electron transport chain (ETC) consists of several of protein complexes situated in the IMM which use the electrons removed by reduced nicotinamide adenine dinucleotide (NADH) and flavin adenine dinucleotide (FADH_2_) from the Krebs cycle to pump protons from the matrix into the intermembrane space, thereby generating a potential gradient across the IMM, which will be used in the final step of OXPHOS to synthesize ATP [56]. In order to function properly, these complexes must be assembled by the folded IMM (mitochondrial cristae) into specifically configured structures [57]. Even under normal conditions, 1–2% of the total oxygen consumed leaks and generates ROS [58]. At least eight mitochondrial sites are able to generate ROS, with complexes I, II, and III being the main contributors [55,59]. Through lipid peroxidation, protein oxidation, and DNA damage, the generated ROS can cause alter mitochondrial function and increase the rate of ROS production, culminating in degeneration of neurons [60,61].

### 3.2. Mitochondria and Cellular Calcium Homeostasis

Calcium is involved in many neuronal functions, such as differentiation, vesicle release and synaptic transmission, or cell death and survival [62,63]. Transient fluctuations in the cytosolic Ca^2+^ act as second messengers. Free cytosolic Ca^2+^ levels are in nanomolar ranges, while extracellular levels are millimolar. Calcium influx occurs through ligand-operated or voltage-gated calcium channels (VGCCs), but Ca^2+^ can also be released from intracellular Ca^2+^ stores, among which the endoplasmic reticulum (ER) has a pivotal role [64]. Agonist binding to inositol 1,4,5-triphosphate (IP3) receptors or to ryanodine receptors (RyRs) causes a release of Ca^2+^ from the ER [65]. However, in order for the increases of cytosolic Ca^2+^ to be brief, calcium must be rapidly cleared through calcium efflux, binding to Ca^2+^-buffering proteins [66], or uptake into the ER or mitochondria [62].

**Figure 1:**
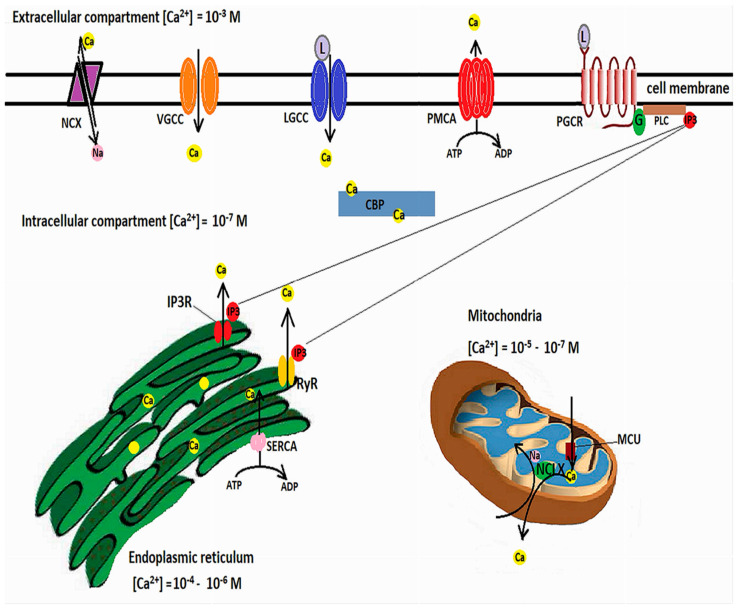
Intracellular calcium homeostasis. Cellular Ca^2+^ influx is mediated by voltage-gated calcium channels (VGCC), ligand-gated calcium channels (LGCC), and, in exceptional circumstances, by reverse functioning of the sodium/calcium exchanger (NCX). In addition, Ca^2+^ can be released from the ER following inositol-1,4,5-triphosphate (IP3) binding to specific receptors (IP3R) or the ryanodine receptors (RyR). IP3 is generated by binding of ligands to plasmalemmal G-protein-coupled receptors, which activates phospholipase C to cleave phosphatidylinositol 4,5-biphosphate, resulting in the second messenger IP3. Excess cytosolic calcium is removed through efflux through the NCX and plasma membrane Ca^2+^ ATPase (PMCA) and uptake into ER by the sarcoendoplasmic reticulum Ca^2+^-ATPase (SERCA). Mitochondria buffers cytosolic calcium through the mitochondrial calcium uniporter (MCU) and extrudes excess Ca^2+^ through the Na^+^/Ca^2+^ exchanger (NCLX). In addition, cytosolic Ca^2+^ binding proteins (CBP) act as signal transducers.

Ca^2+^ efflux is achieved by the plasma membrane Ca^2+^-ATPase, which pumps Ca^2+^ against the concentration gradient while hydrolyzing ATP, and the Na^+^/Ca^2+^ exchanger (NCX), which relies the sodium gradient to extrude Ca^2+^ [67]. ER Ca^2+^ uptake is performed by the ATP-dependent sarco-endoplasmic reticulum Ca^2+^-ATPase (SERCA) [68], while mitochondrial Ca^2+^ uptake is mediated by voltage-dependent anion-selective channel proteins (VDCAs), which mediate Ca^2+^ transfer into the intermembrane space, from where the IMM-located mitochondrial Ca^2+^ uniporter (MCU) further transfers the calcium into the mitochondrial matrix. Increases in mitochondrial calcium activate the ETC dehydrogenases and ATP production [69]. However, calcium overload can alter the mitochondrial membrane potential, open the mitochondrial permeability transition pore (MPTP), and lead to cytochrome c release [70]. As such, mitochondrial Ca^2+^ concentrations must be finely tuned. A mitochondrial Na^+^/Ca^2+^ exchanger located in the IMM, and termed NCLX because it can also able to exchange Li^+^ for Ca^2+^, extrudes Ca^2+^ from the mitochondrial matrix by using the electrochemical gradient of Na^+^ [71], while from the intermembrane space Ca^2+^ is extruded by the Na^+^/Ca^2+^ exchanger 3 and VDACs [72]. Figure 1 illustrates the mechanisms involved in cellular calcium homeostasis.

In controlling the intracellular Ca^2+^ concentrations, mitochondria interact with the ER through mitochondria-associated ER membranes (MAMs) [73], microdomains where the OMM is just 10–100 nanometers apart from the ER [74,75]. These areas are enriched in inositol 1,4,5-triphosphate receptors (IP3Rs) [76] which form functional complexes with VDACs through a chaperone, Grp75 (glucose-regulated protein 75), belonging to the heat shock protein 70 family [77]. IP3R-Grp75-VDAC complexes regulate Ca^2+^ transfer from the ER to mitochondria [74]. The apposition of ER to mitochondria is controlled by phosphofurin acidic cluster sorting protein 2 (PACS2) [78]. Decreased contact sites between ER and mitochondria caused by PACS2 deletion result in mitochondrial fragmentation and apoptosis [79]. PACS2 is functionally linked to phosphatidylserine synthase-1 (PSS1), an enzyme located in MAMs which mediates the transfer of lipids between ER and mitochondria [80]. PACS2 and PSS1 were found upregulated in AD transgenic mice as well as human patients with late-onset AD [74,81]. Several other components of MAMs involved in calcium homeostasis and signaling cascades have been described, such as:

- Bap31 (B cell receptor-associated protein 31), which interacts with the OMM protein Fis1 [82];
- VAPB (Vesicle-associated membrane protein-associated protein B), which interacts with the OMM protein tyrosine phosphatase-interacting protein 51 [83];
- Sig-1R (Sigma non-opioid intracellular 1-receptor 1), a chaperone which binds Grp78; under ER stress conditions, Grp78 dissociates from the ER lipid rafts and activates the unfolded protein response (UPR) [84,85];
- Protein kinase-like endoplasmic reticulum kinase (PERK), which, when activated reduces protein synthesis until the accumulated unfolded protein is cleared [86].

### 3.3. Mitochondrial Dynamics

Mitochondria are dynamic organelles, being able to modulate their number, shape, size, and position in the cytoplasm through a careful balancing of two opposite processes: mitochondrial fusion and fission [87].

Fission is regulated by two proteins: Drp1 (dynamin-related/-like protein 1) and Dnm2 (dynamin 2) [88]. The initial step is wrapping of the endoplasmic reticulum around the mitochondria and reducing the diameter of the latter from 300–500 nm to about 150 nm [89]. The spatial association of replicating mtDNA with the ER-mitochondria contact sites explains the mtDNA distribution in the replicating organelles [90]. Following this step, the cytosolic protein Drp1 is recruited to the already marked constriction site on the OMM and bound to the phospholipid membrane by adaptor proteins such as MFF (mitochondrial fission factor) and mitochondrial dynamics proteins 49 and 51 (MiD49 and MiD51) [91]. Following Drp1 recruitment, a ring-like structure is formed around the mitochondria [92], after which GTP hydrolysis potentiates the constriction of the mitochondrial membrane [87]. The final step is recruitment of Dnm2, a GTPase which assembles at the marked site and completes the fission process [93]. The constriction and division of the IMM is calcium-dependent and occurs at ER–mitochondria contact sites, possibly even before Drp1 recruitment [94].

Mitochondrial fusion is the opposite process, by which the membranes of two mitochondria fuse, giving rise to one single mitochondrion and allowing for sharing of essential components between the two organelles. The process is regulated by two other proteins with GTPase activity, Mfn1 and Mfn2 (mitofusins 1 and 2) for fusion of the OMM, while fusion of the IMM is under control of another GTPase, OPA1 (optic atrophy 1) [95]. After tethering of two mitochondria (mediated through the GTP domains), the two adjacent OMMs increase their contact surface area followed by their fusion due to GTP hydrolysis, subsequent conformational changes, and oligomerization of Mfns [96]. Following OMM fusion, IMM fusion is mediated by IMM-inserted OPA1, which can be cleaved by two membrane-bound metalloproteases, OMA1 and YME1L, resulting in two high molecular weight fragments (L-OPA1) and three shorter fragments (S-OPA1) [97]. The interaction of L-OPA1 with cardiolipin, inserted in the IMM, is crucial for driving membrane fusion [98]. The balance between OMA1 or YME1L cleavage of OPA1 regulates mitochondrial fission [99]. Stimulation of OXPHOS induces YME1L cleavage of OPA1 and mitochondrial fusion, while OPA1 cleavage by OMA1 is a stress response, and may induce mitochondrial fragmentation as well [87].

Several post-translational modifications also regulate mitochondrial dynamics. Drp1 phosphorylation can stimulate either fission or fusion depending on the phosphorylation site [100,101]. Phosphorylation of MFF increases Drp1 recruitment, and subsequent mitochondrial fission [102], while ubiquitination of acetylated Mfn1 promotes its proteasomal degradation [103]; phosphorylation of Mfn1 by ERK inhibits mitochondrial fusion, promoting apoptosis [104]. Specific nutrient states also indirectly regulate the balance between these two processes. While starvation leads to fused and elongated mitochondria, a nutrient-rich environment is accompanied by fragmented mitochondria [105,106].

### 3.4. Autophagy

Autophagy is another essential step in maintaining the balance between protein synthesis and clearance, organelle biogenesis, and degradation, thereby promoting cellular health [107]. Based on the way in which the targeted cargo is conveyed for degradation to lysosomes, autophagy can be classified into [107,108]:

- Macroautophagy, in which the autophagosome, a double-membraned vesicle, forms and fuses with lysosomes after which their content is degraded by the acidic hydrolases of the lysosomes;
- Microautophagy, a process during which lysosomes wrap around various cytosolic compounds which are degraded after the involution of the membrane [109];
- Chaperone-mediated autophagy, a process during which chaperones bind to damaged proteins and to receptors on the lysosomal membrane, followed by translocation of the protein into the lysosome for degradation [110].

Autophagy for damaged mitochondria is also known as mitophagy. The first step in mitophagy initiation is the formation of an isolation membrane, the autophagosome, believed to derive from MAMs, membranes of the ER, or plasma membrane [111,112], followed by activation of the pre-initiation complex, containing ULK1 (Unc-51-like kinase 1, Atg 13 and 101 (autophagy-related proteins), and FIP200 (focal adhesion kinase family interacting partner 200) [113]. The pre-initiation complex recruits class III phosphatidylinositide 3-kinase (PI3K), beclin1, Atg 14, autophagy, and beclin 1 regulator (AMBRA1), as well as vascular protein sorting 34 and 15 (Vps 34 and 15) to produce phosphatidylinositol 3-phosphate (PI3P). Pi3P is also known as the initiation complex [114]. After activation, both complexes translocate to the nucleation site of the phagophore [108]. PI3P is recognized and interacts with other IM-located proteins, such as WD repeat protein interacting with phosphoinositide (WIPI) and FYVE domain containing proteins [115] and leads to a series of conjugations of Atgs on the phagophore, culminating in cleavage of pro-LC3 (light chain 3) by Atg4 to LC3-I, further transformed by phosphatidylethanolamine to LC3-II, and leading to elongation and closure of the isolation membrane [116,117]. Υ-aminobutiric acid type A-receptor-associated protein (GABARAP) and GABARAP-like 1 protein (GABARAPL1) are believed to play similar roles with LC3 in autophagosome expansion [118]. The signaling protein mTOR (mammalian target of rapamycin) strongly modulates autophagy. Inhibition of mTOR, as occurs in starvation, dephosphorylates and activates Atg13, igniting the mitophagy process [119]. When growth factors and cellular nutrients are abundant, mTOR phosphorylates Atg 13, preventing its binding to ULK1 and recruitment of FIP200 [107]. Fusion of autophagosomes to lysosomes is mediated by Rab7 and LAMP-2, a lysosomal transmembrane protein [120,121]. After fusion, lysosomal enzymes, mainly cathepsins, degrade the autophagosomal content [122].

**Figure 2:**
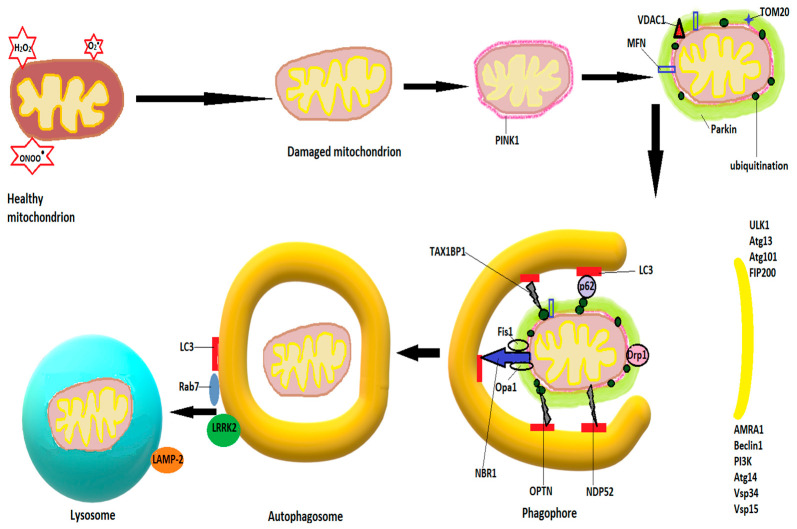
Schematic diagram of mitophagy. The mitophagy induction process activates PINK1, which accumulates on the OMM and recruits and phosphorylates Parkin. The latter ubiquitinates OMM proteins, such as mitofusins 1 and 2 (Mfn), voltage-dependent anion channels 1 (VDAC1), and TOM20 (translocase of the outer mitochondrial membrane 20). Autophagosome formation starts with activation of the pre-initiation complex, containing ULK1 (Unc-51-like kinase 1), Atg 13 and 101 (autophagy-related proteins), and FIP200 (focal adhesion kinase family interacting partner 200), and continues with recruitment of class III phosphatidylinositide 3-kinase (PI3K), beclin1, Atg 14, AMBA1 (autophagy and beclin 1 regulator), and Vps 34 and 15 (vascular protein sorting), resulting in the production of phosphatidylinositol 3-phosphate (PI3P). The ubiquitinated proteins recruit autophagy adaptor proteins, such as neighbor BRCA1 gene (NBR1), optineurin (OPTN), Tax-1 binding protein (TAX1BP1), nuclear dot protein 52 (NDP52), or sequestosome-1, which interact with autophagosome proteins like GABARAP or LC3 to mediate autophagosome formation. Fusion of the autophagosome with lysosomes is mediated by LC3, Rab7, LRRK2, and LAMP-2 (see text).

In non-receptor mediated mitophagy, the mitophagy induction process activates PTEN-induced kinase 1 (PINK1), which accumulates on the OMM and recruits and phosphorylates Parkin [123,124]. Parkin accumulates on the OMM and ubiquitinates OMM proteins, which leads to increased activity of PINK1 and more Parkin recruitment [113]. Among the proteins ubiquitinated by Parkin are voltage-dependent anion channels 1 (VDAC1), Mfn1 and Mfn2, as well as TOM20 (translocase of the outer mitochondrial membrane 20), which govern mitochondrial fusion. Ubiquitination of Mfn1/2 will block the fusion process and allow the isolation of the small and damaged mitochondria [125]. The ubiquitinated proteins recruit autophagy adaptor proteins, such as OPTN (optineurin), NBR1 (neighbor BRCA1), TAX1BP1 (Tax-1 binding protein), NDP52 (nuclear dot protein 52), or sequestosome-1, which interact with autophagosome proteins like GABARAP or LC3 through LC3 interacting regions (LIR) to mediate autophagosome formation and fusion with lysosomes [126,127]. Figure 2 shows schematically the mitophagy process.

However, Parkin-independent pathways of mitophagy exist as well [113], such as receptor-mediated mitophagy. The most studied proteins involved in receptor-mediated mitophagy are AMBRA1, FUNDC1 (FUN14 domain-containing protein 1), NIX (Nip3-like protein), and BNIP3, located on the OMM, as well as cardiolipin and prohibitin 2 (PHB2) on the IMM [113]. These receptors can bind to LC3 in a Parkin-independent manner [128] and induce mitophagy. Their transcription can be activated under various conditions. For example, transcription of BNIP3 and NIX are activated by hypoxia via hypoxia inducible factor 1 alpha (HIF1α) [129], which, after phosphorylation have a high binding affinity for LC3 [130]. Dephosphorylation of FUNDC1 by hypoxia facilitates its binding to LC3 [131].

In recent years, researchers have shown that mitochondria can be extruded from cells and taken up by endocytosis or phagocytosis by neighboring cells, where they ultimately undergo mitophagy, a phenomenon termed transcellular mitophagy [113,132]. It is reasonable to assume that transferring mitochondria back to the cell soma from dendrites or axons would be energetically unfavorable, which is why neurons release mitochondria at synapses to be degraded by glial cells [133]. In turn, glial cells can transfer mitochondria to neurons and protect the latter from hypoxia and energetic failure [134]. The precise pathways for mitochondrial transfer are still investigated, but some studies suggested an important role for a protein connecting mitochondria to cytoskeletal motor proteins, namely MIRO1 [135] while others showed involvement of astrocytic GFAP (glial acidic fibrillary protein) and neuronal UCP2 (uncoupling protein 2) [136].

Another way of disposing of damaged mitochondria has been identified in 1992 [137] and named mitoptosis. It enables the cells to degrade mitochondria without opening of the MPTP and igniting apoptosis [113]. Mitoptosis is likely activated by mitochondrial membrane depolarization, damage of the mitochondrial DNA (mtDNA), and ROS [113]. The exact mechanisms of mitoptosis require further study, but several situations have been described, such as swelling and fragmentation of cristae followed by cytoplasmic extrusion of cristae fragments through bursting of the OMM [138], or deterioration of cristae through coalescence of the IMM with preservation of intact OMM [138].

ROS play a key role in regulation of autophagy and mitophagy. One pathway, mentioned above, is the mTOR pathway. An amino acid-rich environment leads to translocation of mTOR complex 1 (mTORC1) to the lysosomal surface, where it interacts with Rheb and activates mTOR [139], while in starvation mTOR colocalizes with LC3 and initiates autophagy [140]. Considering that mTOR oxidation inhibits its activity, it is very likely that ROS regulate this step [141]. Further, S-nitrosation of IκB kinase β and JNK1 by nitric oxide inhibits their activity, which, in turn, prevents mTOR inactivation and release of Beclin from the Beclin-Bcl-2 complex [93,142].

Another pathway is the beclin-1-class III PI3K complex, with a series of cofactors such as AMBRA1, Bax-interacting factor 1 (Bif-1), or Rubicon (RUN domain- and cysteine-rich domain-containing beclin-1-interacting protein) [143]. Other regulators of autophagy include IP3 receptor, AMPK (5′-AMP-activated protein kinase) and DAPK (death-associated protein kinase) [144,145].

ROS and reactive nitrogen species induce post-translational protein modifications which also regulate the activity of transcription factors. For example, in the Nrf2 (nuclear factor-erythroid 2-related factor 2)/Keap1 (Kelch-like enoyl-CoA hydratase-associated protein 1) pathway, modifications of Keap1 lead to release of Nrf2, which binds to ARE (antioxidant-response element) and translocates to the nucleus, where it activates the transcription of antioxidant enzyme genes and proteins, such as p62 or p53. While p62 activates autophagy [146,147], p5 is linked to both autophagy-inhibiting and -promoting genes through TIGAR (tumor protein 53-induced glycolysis and apoptosis regulator) and DRAM (damage-regulated autophagy modulator), respectively [148,149]. In turn, impaired regulation of autophagy leads to increased oxidative stress and accumulation of ubiquitinated proteins, the latter causing mitochondrial dysfunction and further augmenting ROS generation in a feed forward loop [93].

## 4. The Brain and Oxidative Stress

Oxygen is crucial for proper cellular functioning, being involved in the generation of ATP [61]. Unfortunately, due to the univalent metabolic reduction status of oxygen, with the two lone electrons of oxygen spinning in parallel, it can accept only one electron at a time [150], leading to the generation of species having one unpaired electron which can exist on their own, defined by Halliwell as free radicals [151]. The oxygen derivatives are either free radicals, such as the superoxide anion (^•^O_2−_), hydroxyl radical (HO^•^), hydroperoxyl radical (HO_2_^•^), and peroxyl radicals (ROO^•^), or non-radicals which can be transformed into radicals, such as hydrogen peroxide (H_2_O_2_) [61,152]. Redox signaling is extensively used in the brain [153], being involved in signal transduction and gene transcription. For example, NADPH oxidases (NOXs) regulate hippocampal long-term potentiation [154] and NOX2-derived superoxide and hydrogen peroxide regulate hippocampal progenitor cell growth in adult brain via the phosphatidyl inositol 3 kinase (PI3K)/Akt signaling pathway [155]. Similarly, NOX-derived H_2_O_2_ have beneficial roles in axonal regeneration and axonal pathfinding during wiring of the developing brain [156,157]. As a consequence of hypoxia, mitochondria-derived superoxide-induced signaling leads to adaptive responses [158]. To counterbalance the possible deleterious effects of excessive free radicals, the biological systems have a series of antioxidant defenses, which can be divided into enzymatic ones (superoxide dismutases, catalase, glutathione peroxidases, glutathione transferases, thioredoxins, and peroxiredoxin) and non-enzymatic defenses, such as vitamins A, C, E, beta-carotene, or glutathione [159]. Whenever the rate of free radical production exceeds the biological system’s ability to neutralize them, oxidative stress ensues.

### 4.1. Vulnerability of the Brain to Oxidative Stress

The nervous system is very sensitive to oxidative stress, due to a series of reasons [153,160,161,162]:

- Action potentials cause calcium influx and raise the intracellular calcium concentration from approximately 0.001 μm to roughly 100 μm [163]. High intracellular Ca^2+^ activates nNOS (neuronal nitric oxide synthase) and leads to NO (nitric oxide) formation [164], which binds to cytochrome c oxidase and inhibits mitochondrial respiration [165]. Mitochondria attempt to buffer intracellular calcium, but the subsequent calcium overload causes prolonged opening of the MPTP and inhibits ATP generation, inducing apoptosis [166].
- The brain has very high energy demands to maintain the ionic gradients and support synaptic transmission [19] and relies mainly on synaptic mitochondria for the generation of required energy [167]. For example, neurotransmitter vesicle release requires 1.64 × 10^5^ ATP/s/vesicle [19].
- The brain has low antioxidant defenses. Neuronal cells have 50 times less catalase than hepatocytes [168], while cytosolic glutathione is about 50% lower in neurons compared with other cells [153], and this might diminish peroxiredoxin activity [169].
- Microglia, the immune cells of the brain, are activated by H_2_O_2_ [170] and produce superoxide via NADPH oxidase isoforms, needed for bacterial killing [171].
- The metabolism of neurotransmitters, such as dopamine metabolism via monoamine oxidases, generates ROS [153,172].
- Neurotransmitters, such as dopamine, serotonin, or adrenaline, can auto-oxidize and generate superoxide [173,174].
- The brain is enriched in redox active transition metals, such as Cu^+^ or Fe^2+^ [175]. Iron is a catalyzer in the hydroxyl radical-generating Fenton reaction, and also catalyzes peroxyl and alkoxyl radical generation, thereby contributing to ferroptosis, a form of cell death dependent on lipid peroxidation and Fe^2+^ [176]. Cu^+^ is a co-factor for Cu/ZnSOD and is important for cell signaling [177,178] but enhances copper-catalyzed Fenton reaction [175].
- The brain is particularly rich in cholesterol, which may undergo auto-oxidation [179] and brain cells have a higher membrane surface/cytoplasmic volume ratio, cellular membranes being rich in polyunsaturated fatty acids (PUFA), which are highly susceptible to peroxidation through free radical attack [153].
- Brain development and plasticity relies on non-coding RNAs (long non-coding RNAs and microRNAs) [180], but these molecules lack protective histones and are easily oxidized [181]. Oxidized messenger RNA results in truncated and mutated proteins, prone to misfolding [182].

### 4.2. Sources of Free Radicals

Excess free radicals can be generated from many sources.

#### 4.2.1. Mitochondria and Oxidative Stress

Mitochondria are traditionally regarded as main sources of ROS. At least 10 potential sources of ROS production have been identified [183] but complexes I (NADH dehydrogenase) and III (ubiquinone cytochrome c reductase) of the ETC [184] are the most important ones. Transferring electrons to coenzyme Q or ubiquinone by complexes I and II results in ubiquinol (reduced ubiquinone, QH_2_), which will regenerate coenzyme Q via semiquinone anion (^•^Q^−^), an unstable intermediate which can transfer electrons to molecular oxygen, resulting in superoxide formation [60]. Being a non-enzymatic reaction, higher metabolic rates lead to increased superoxide production [185]. Superoxide is highly unstable and is transformed by the mitochondrial superoxide dismutase 2 (SOD2, manganese SOD) and the cytosolic SOD1 (copper zinc SOD) into the more stable hydrogen peroxide (H_2_O_2_). The latter can exit the IMM through aquaporin channels and diffuse through the OMM into the cytoplasm, where it serves for redox signaling, or is further reduced to water by catalase, glutathione peroxidases, and peroxiredoxins [186,187,188,189]. Other mitochondrial components contributing to ROS formation include monoamine oxidase, glycerol phosphate dehydrogenase, α-ketoglutarate dehydrogenase, and p66shc [60,183,190].

Mitochondrial ROS production is subject to variations induced by metabolic factors. For example, the NADH/NAD^+^ ratio affects the rate of ROS generation, which increases almost linearly with NADH reduction [191]. Succinate levels can fluctuate even in normal conditions between 0.3 and 1 mM [192], with increased succinate concentrations strongly increasing mitochondrial ROS generation [183,193]. The rate of ROS production is also influenced by the mitochondrial membrane potential [183]. Active mitochondrial phosphorylation of ADP or mitochondrial calcium uptake decreases the membrane potential, which influences the redox potential of the ETC and decreases ROS production [194]. Oxygen deprivation or ischemia significantly increase mitochondrial ROS generation [195], although this effect may not be attributed to mitochondria per se, but rather to signaling pathways triggered by hypoxia [196].

#### 4.2.2. NADPH Oxidase as a Source of ROS

NADPH oxidase (NOX) was first described in phagocytes [197], after which seven NOX genes have been identified: NOX 1–5 and DUOX 1 and 2 [198]. The brain expresses mainly NOX2, as well as NOX4, both being described in the cortex and CA1 hippocampal areas [199]. The NOX2 enzyme complex has a membrane-bound cytochrome b558, several cytosolic proteins, and the Rac G-protein. Following phosphorylation of the cytosolic proteins and activation of Rac, the enzyme translocates to the membrane and forms active NOX2 with cytochrome b558 [200]. Further, NOX2 transfers protons across the membrane and leads to superoxide generation [198]. NOX4 produces mainly H2O2, used as a second messenger for cell proliferation and differentiation [201]. NOX has been described in neurons, astrocytes, and microglia [202], while at the cellular level, NOX isoforms localize to the endoplasmic reticulum, nucleus, plasma membrane, and mitochondria [203,204]. The ROS generated by activated NOX can depolarize the mitochondrial membrane and, together with calcium, can lead to opening of the MPTP [205] as well as activate phospholipase C with subsequent changes in membrane structure [198].

#### 4.2.3. Monoamine Oxidase as a Source of ROS

The monoamine oxidases (MAO A and B) are flavoenzymes located on the OMM which catabolize amine neurotransmitters, such as serotonin, epinephrine, and dopamine [206]. MAO A is expressed in neurons, while MAO A and B can be found in glial cells [198]. They use FAD to break down monoamines, a process during which aldehydes are produced, while H_2_O_2_ results from the FAD-FADH2 cycle [198].

#### 4.2.4. Peroxisomes and ROS Production

Although the major metabolic peroxisomal process leading to H_2_O_2_ generation is β-oxidation of free fatty acids [60], several other peroxisomal enzymes, such as xanthine oxidase, D-aspartate oxidase, acyl CoA oxidases, D-amino acid oxidase, urate oxidase, or L-α-hydroxy oxidase, can produce a variety of ROS, such as superoxide, hydrogen peroxide, nitric oxide, or hydroxyl radicals [207].

#### 4.2.5. Exogenous Sources of ROS

In addition to the multiple sources of endogenous ROS, exogenous ROS can augment oxidative stress. The most common sources are water and air pollution, ultraviolet light exposure, alcohol and tobacco smoke, pesticides, industrial solvents, unhealthy diets (with smoked meat, high-fat diet), exposure to heavy metals or transition metals (Fe, Cr, Co, Cu, Hg, Pb, As), as well as certain drugs, such as Doxorubicin, Bleomycin, Metronidazole, or even Paracetamol [60].

### 4.3. Targets of ROS

The highly reactive free radicals damage proteins, lipids, and nucleic acids [152].

#### 4.3.1. Proteins and ROS

Proteins can be oxidized by radicals, such as superoxide, hydroxyl radical, peroxyl, hydroperoxyl, or alkoxyl radicals, as well as by non-radical species or singlet oxygen [208]. Oxidation of amino acids such as lysine, arginine, proline, or threonine leads to carbonyl derivatives, used as markers of oxidative stress [209]. Methionine and cysteine, as sulphur-containing amino acids, are very susceptible to oxidation, leading to disulphides and methionine sulphoxide [210]. Protein oxidation leads to protein–protein cross linkages, altering of function, loss of enzymatic activity, and functional modifications of receptor and transport proteins [211]. Moreover, hydrogen peroxide and hydroxyl radicals inhibit glutamate uptake by astrocytes, augmenting excitotoxicity [212]. As a consequence, these altered proteins must be cleared, either by the autophagy-lysosome pathway or by the ubiquitin-proteasome system [213,214]. The ubiquitin-proteasomal system (UPS), the main degradation pathway of ubiquitinated misfolded proteins and short-lived signaling molecules, contains a 19S subunit and a catalytically active 20S core [215]. The regulatory cap with chaperone proteins unfolds the target protein, removing the ubiquitin tag in an ATP-dependent process, after which the target protein is fed into the catalytic core and is degraded by the proteasomal enzymes. Under severe stress, the UPS is overwhelmed, and the autophagy-lysosome pathway compensates for the increased protein damage [107]. A series of autophagy receptors, such as p62, NDP52, or NBR1 recognize the ubiquitin moiety and target tagged proteins to the autophagosome by binding to Atg8/LC3 [216,217]. FOXO3 (forkhead box O3) is a transcription factor activated by oxidative stress, which regulates the transcription of genes involved in proteasomal as well as autophagic protein degradation [218]. In addition, Parkin, ubiquitin ligases, and CHIP (C-terminus of Hsc70-interacting protein) contribute to both proteasomal and autophagosomal degradation of proteins [107,219]. The relative levels between BAG (Bcl-2 associated atanogene) 1 and BAG3, co-chaperone proteins, direct the cellular protein degradation pathway towards the proteasomal or phagosomal one [220].

#### 4.3.2. Lipids and ROS

Free radicals or non-radical oxidative species attack the C-C double bonds of lipids, which is why polyunsaturated fatty acids are very vulnerable to oxidative attack [221]. In the initial phase, a free radical interacts with a methylene group in the fatty acid and generates a lipid radical by dissociating a hydrogen atom [222]. Further, the lipid radicals react with molecular O_2_ to form peroxyl radicals (ROO^•^) [223], which initiate a chain of self-sustained reactions amplifying the process. This results in cyclic peroxides and hydroperoxides, which can further be degraded to aldehydes, the final products being malondialdehyde (MDA), hydroxynonenal (HNE), and acrolein [224]. The process is terminated either through interaction of lipid radicals with lipid peroxides, resulting in non-reactive stable species [221], or through intervention of endogenous or exogenous antioxidants (vitamins C and E) [222,225].

In low concentrations, 4-HNE plays important homeostatic roles by acting as a signaling molecule and modulating gene expression by inducing post-translational protein modifications. The most common targets are thiol residues [225]. By interacting with cysteine thiols in Keap1, 4-HNE leads to release of Nrf2, which after translocating to the nucleus activates the expression of ARE genes such as glutathione-S-transferase, NADPH-dependent quinone reductase, or heme oxygenase-1 [226]. Another target of 4-HNE is NF-κB, a transcription factor for pro-inflammatory cytokines, normally maintained quiescent by binding to IκBα (inhibitor kappa B). Under cellular stress, IκB kinase phosphorylates IκBα facilitating the release of NF-κB, a transcription factor which mediates the transcription of antiapoptotic Bcl-2 proteins and of inflammatory cytokines, such as interleukin-6 (IL-6). 4-HNE inhibits IκB kinase, thus preventing IκBα phosphorylation and NF-κB nuclear translocation [227].

However, as a consequence of membrane lipid peroxidation, the membrane alters its permeability, increases its rigidity, and may even loose integrity [228]. In addition, lipid peroxidation products are involved in complex signaling pathways. Intracellular accumulation of 4-HNE can lead to apoptosis through both the intrinsic and extrinsic pathways [225]. 4-HNE increases the expression of p53, followed by activation of p21, JNK, Bax, and caspase 3 [229], leading to caspase-mediated apoptosis. In addition, 4-HNE initiates the binding of the death-associated protein Daxx to the intracellular surface of Fas [230], thereby being involved in modulation of the extrinsic pathway of apoptosis through the down-stream signaling proteins ASK1 and JNK [225]. Moreover, high levels of HNE can form conjugates with JNK, responsible for histone modification and facilitating of nuclear translocation [231] or can activate JNK through SPKK1 (stress-activated protein kinase kinase-1) activation [232]. Similarly, HNE can activate ERK via activation of MEK1/2 and p38MAPK (mitogen activated protein kinase) [233,234].

#### 4.3.3. DNA

ROS can induce many types of DNA damage, such as base modification, deoxyribose modification, single strand breaks (SSBs) and double strand breaks (DSBs), DNA cross-links, or abasic sites [235]. Oxidized DNA is probably the most common DNA lesion in neurons [236]. As under normal conditions ROS can cause up to 50,000 DNA lesions/cell/day [237], delays in repair of DNA damage can cause genomic instability and induce signaling cascades leading to cell death [238]. The cells have various repair mechanisms, such as base excision repair (BER), mismatch repair (MMR), nucleotide excision repair (NER), and single- (SSBR) and double-strand break repair (DSBR) mechanisms [239].

Oxidized DNA bases are removed mainly by the BER/SSBR mechanism and involve the removal of the damaged base by a specific DNA glycosylase (DG), incision of the abasic site by an AP-endonuclease (APE1), filling of the resulting gap by a DNA polymerase, and sealing of the damaged DNA strand by a DNA ligase [240]. Several DGs have been identified, such as uracil-DNA glycosylases (UDGs), thymine DNA glycosylase (TDG), or oxidized base-specific DGs, such as 8-oxoGuanine (8-oxoG) DNA glycosylase (OGG1) [241,242]. OGG1-1a is involved in nuclear DNA repair, while OGG1-2a contributes to mitochondrial DNA repair [243]. APE1 cleaves the sugar-phosphate backbone of DNA at the abasic site. Phosphorylation of the enzyme, as happens after 1-methyl-4-phenylpyridinium (MPP+) exposure, reduces its enzyme activity and results in accumulation of damaged DNA in neurons [244].

Mitochondrial DNA is even more prone to oxidative damage because the mitochondrial genome is situated close to the IMM, the site of mitochondrial ROS generation, and because it lacks protective histones [245]. As mitochondrial DNA quality control is crucial for the communication with the nucleus, mitochondria contain antioxidant and DNA repair enzymes, such as OGG1 and MUTYH (mutY DNA glycosylase) [246]. Mitochondrial dysfunction, as happens after mtDNA deletions, can lead to activation of NF- κB through a calcineurin-dependent signaling pathway [247] and can, in turn, be induced by various signaling molecules. For example, PPAR-γ coactivator 1 and SIRT1 regulate mitochondrial function by activating the expression of mitochondrial transcription factor A (TFAM) [248].

In addition to increased oxidative damage caused by oxidative stress, defective DNA repair mechanisms have been described in neurodegenerative diseases, such as AD or PD [235]. Dopaminergic neurons of PD patients showed upregulation of mitochondrial OGG1 and higher levels of phosphorylated APE1 [249]. Neurons from AD patients had higher levels of oxidized DNA bases in nuclear as well as mitochondrial DNA [250], exhibited a decrease of OGG1 activity [251] and lower levels of UDG [252] in comparison with neurons from healthy individuals.

#### 4.3.4. RNA and Oxidative Damage

RNA is more abundant, accounting for 80–90% of the nucleic acid in cells [253] and is more vulnerable to oxidative damage due to its single stranded structure, proximity to the mitochondria, lack of oxidized RNA repair mechanisms, and less protection from proteins as compared to DNA [60,254]. The functions of RNA molecules in the cell are diverse, comprising the ribosomal RNA (rRNAs), which together with the transfer RNA (tRNA) and messenger RNA (mRNA) is responsible for protein synthesis, microRNAs (miRNAs), which are post-translational regulators of gene expression, and small nuclear and nucleolar RNAs. Traditionally, mRNA was regarded as the coding RNA [255]. The non-coding RNAs play important roles in mRNA splicing regulation and mRNA translation [256].

The most aggressive radical is the hydroxyl radical, produced in the Fenton and Haber–Weiss reactions [257]. It reacts with guanine to form 8-hydroxyguanosine (8-OHG), one of the most commonly used biomarkers of RNA oxidation [253]. The oxidative attack-induced damage to RNA leads to modified bases and nucleosides, cleavage and fragmentation of tRNA, aminoacylation, or defects in codon–anticodon pairing [258] and cause direct RNA strand breaks [259], base mismatches on tRNAs, translation errors [182], and disordered protein synthesis [260] with significant impact on cell viability. Although cells have several mechanisms to degrade altered transcripts, such as the nonsense-mediated mRNA decay (NMD) [261], these mechanisms tend to be overwhelmed with aging. As such, altered misfolded proteins accumulate [262].

## 5. Selective Neuron Vulnerability in Neurodegenerative Diseases

Although the many pathways leading to increased ROS generation and their consequences apply to all cells, each neurodegenerative disease leads to degeneration of particular groups of neurons, which led researchers to look for explanations for this selective vulnerability of neuronal populations.

### 5.1. Selective Neuronal Vulnerability in Alzheimer’s Disease

The memory loss and cognitive decline characteristic of AD are caused by atrophy of the entorhinal cortex (EC), mainly of neurons in layer II (ECII), and hippocampus, particularly the CA1 region [72]. Research has shown that neurons in these areas have high energetic demands and are very sensitive to decreased oxygen and glucose supply [263]. In addition, CA1 and EC II pyramidal neurons are glutamatergic and thereby more vulnerable to NMDA excitotoxicity and the damaging effects of increased intracellular calcium concentrations [264], as opposed to neocortical inhibitory interneurons, which have high levels of Ca^2+^-binding proteins [265]. A series of molecular hallmarks of the vulnerable neurons have been recently described. For example, ECII pyramidal neurons have an impaired activity of a regulator of tau splicing, likely linked to disturbed microtubule dynamics [266], which may facilitate tau spreading to other brain regions via CA1 neurons [267]. Selectively vulnerable excitatory neuron subpopulations were shown to express RORB (RAR-related Orphan Receptor B), while also exhibiting differences in the expression of genes encoding synapse- versus axon-localized proteins, subunits of the potassium channels, G-protein signaling molecules and neurotransmitter receptor signaling molecules [268]. However, the connections between RORB expression, accumulation of phosphorylated tau, and neural degeneration remain to be further characterized.

### 5.2. Selective Neuronal Vulnerability in Parkinson’s Disease

The motor symptoms of PD are caused by the loss of nigral dopaminergic neurons leading to dopamine depletion in the dorsal striatum [269]. Structurally, these neurons have very long and branched axons (up to 4.5 m), being connected to a large number of neurons (up to 2.4 million synapses) [270,271], which requires a high density of axonal mitochondria and challenges mitochondrial bioenergetics [272].

At the molecular level, nigral dopaminergic neurons have low Ca^2+^-buffering capacity despite high activity-dependent Ca^2+^ loads, which increase OXPHOS and ROS generation [273], and may promote mtDNA damage [274]. In addition, the metabolism of dopamine itself leads to ROS generation and causes accumulation of mtDNA deletions [275].

### 5.3. Motor Neuron Vulnerability in Amyotrophic Lateral Sclerosis

The pathological hallmark of ALS is degeneration of upper and lower motor neurons, which both have very long axons and, thus, depend on proper mitochondrial function and trafficking [72]. The large motor unit size imposes high energetic demands on spinal motor neurons to maintain neurotransmission and muscle contraction [276]. The relative preservation of motor neurons in the oculomotor, trochlear and abducens nuclei may be linked to the small number of innervated muscle fibers (up to five muscle fibers as opposed to at least 300 fibers innervated by spinal motor neurons) [277], the particular grape-like structure of the neuromuscular junction [278], as well as the high Ca^2+^ buffering capacity of these neurons [279].

## 6. Oxidative Stress in Neurodegenerative Diseases

Research has increasingly shown the presence of oxidative stress markers in the nervous system of patients having neurodegenerative diseases, but the molecular pathways are just beginning to be elucidated. The description of genetic defects leading to AD, PD, HD, or ALS helped in identifying downstream effects of the mutant proteins, similar pathways being subsequently demonstrated in idiopathic forms of the diseases. However, the pathophysiology is still incompletely elucidated, despite accumulated knowledge having already led to therapeutic attempts.

### 6.1. Oxidative Stress in Alzheimer’s Disease

Familial cases of AD with mutations of Presenilin 1 (PS1) and 2 (PS2) have linked AD pathogenesis with disturbed calcium homeostasis [280]. PSs interact with the ryanodine receptors [281] and influence ER–mitochondria coupling [282]. Indeed, high MAM numbers have been described in animal models of AD and in fibroblasts or brain tissue of AD patients [283]. In addition, Aβ aggregates can mediate Ca^2+^ transfer from ER to the mitochondria through the MCU [284], while tau inhibits mitochondrial calcium efflux [285]. Moreover, Aβ can form calcium-permeable channels in membranes [286,287], while tau can form non-selective ion channels in lipid bilayers [288]. The deleterious effects of increased mitochondrial calcium in inducing mitochondrial dysfunction and oxidative stress have been described in the previous sections. In astrocytes, Aβ interacting with the calcium sensing receptors (CaSRs) induces their downregulation, leading neighboring neurons to secrete newly synthesized Aβ, nitric oxide, and peroxynitrite [289].

In the mitochondria, one of the earliest alterations linked to AD is lipoxidation of ATP synthase with consequent reduction of its function, described in EC neurons as early as Braak stages I-II [290,291]. Located inside the IMM, inserted in a PUFA-rich lipid bilayer and close to the mitochondrial matrix, the enzyme is an easy target for free radicals generated by complexes I and III [292]. In addition to energetic failure, ATP synthase modification further increases ROS production with subsequent potentiation of oxidative modifications of biological molecules [293]. In this cascade, most modified lipoxidation products are involved in energy metabolism, increasing energy failure, followed by proteins involved in neurotransmission, antioxidant defenses, and ion channels, as shown in Table 1.

The communication between mitochondria and nucleus, and the import of nuclear-coded mitochondrial subunits are impaired by the bioenergetic defects and dysfunctional mitochondrial proteins, leading to altered expression of regulatory and structural mitochondrial complexes and enzymes [299]. The lipid metabolism is also altered, with changes in lipidomic profiles described in AD, especially in the EC [300]. The oxidative damage to PUFAs leads to the reduction of these fatty acids in the lipid rafts in EC from early stages of AD [301], with consequences on membrane thickness, fluidity, curvature, as well as activity of membrane-bound proteins, which favor amyloidogenic processing of amyloid precursor protein. It appears that mitochondrial oxidative stress, altered lipid metabolism, lipid peroxidation, and bioenergetic defects are all part of a self-sustained loop which contributes to augmentation of the altered mitochondrial dynamics, mitochondrial trafficking, impaired mitophagy, and impaired ER–mitochondrial interaction [290].

The intracellular build-up of amyloid beta (Aβ) and phosphorylated tau induces much of the altered mitochondrial dynamics, expressed as excessive mitochondrial fragmentation, with increased number and decreased size of mitochondria [302]. Both Aβ and phosphorylated tau increased the GTPase activity of Drp1, which leads to excessive mitochondrial fragmentation [303,304]. In vitro, mutant APP cellular lines exhibited high concentrations of mRNA and proteins of mitochondrial fission and diminished levels of mRNA and proteins of mitochondrial fusion [305]. Phosphorylated tau has also been shown to downregulate Opa1 and upregulate Mfn1 and Mfn2 [306]. In addition, cytoplasmic accumulation of Aβ leads to depletion of Parkin and PINK1 levels, thereby interfering with the mitophagy pathway. Defective mitophagy results in accumulation of autophagic vacuoles in the neuronal soma and dysfunctional neurites [307], a process potentiated by the particularities of mitophagy in neurons, where mature lysosomes are concentrated in the cell body whereas mitochondria extend along the axons and dendrites of neurons, making neuronal mitophagy a slower process [308]. In addition, Aβ oligomers interact with autophagic vacuoles in the distal parts of axons, leading to inhibition of mitochondrial axonal transport [309]. In addition, in laboratory models, mutations in the PS1 gene altered lysosomal acidification [310] and led to diminished expression of autophagy-related genes through the ERK/CREB signaling pathway [311].

Protein tau has also important contributions to the protection of the cellular genome in physiological conditions, by binding chromatin [236]. The hyperphosphorylated state of tau interferes with this function, creating the premises for infliction of more oxidative DNA damage and longer time needed to repair these DNA lesions [236].

One must not overlook the role of microglial activation and chronic inflammation in the pathogenesis of AD. Microglia, the innate immune macrophage-like cells of the nervous system, account for about 10% of the cellular population in healthy adult brain [312]. In AD, microglia have increased expression of complement receptors leading to upregulation of the NF-κB signaling pathway, Aβ-activated Fc receptors (which induces the expression of MIP-macrophage inflammatory protein-1α), and increased expression of scavenger receptors A-1 and B (SCARA and SCARB) [313,314]. Binding of Aβ to SCARB-2 activates microglia, to produce proinflammatory cytokines, chemokines, and ROS [315]. Increased levels of tau, by inducing the expression of toll-like receptors, also lead to the release of pro-inflammatory cytokines IL-1β, IL-6, IL-8 through the NF-κB signaling pathway [316]. Although inflammation activates autophagy in cells [108], since impairment of autophagy affects microglial cells as well, the chronic inflammatory microglial phenotype supplementary contributes to neuronal damage in AD [317].

### 6.2. Oxidative Stress in Parkinson’s Disease

PD is the second most common neurodegenerative disease, exhibiting both cognitive and neuromuscular impairments. Although most cases are sporadic, several mutations have been described in familial and early-onset forms of PD, such as mutations in PARK1 (encoding PINK1), PARK2 (encoding for Parkin), PARK1/4 (α-synuclein), PARK7 (DJ1), PARK8 (LRRK2), PARK9 (ATP13A2) PARK17 (Vsp35), FBX07, GIGYF2, or HTRA2, highlighting the involvement of the ubiquitin protein degradation pathway, oxidative stress, cell survival pathway, mitochondrial function, and apoptosis in PD pathogenesis [113,318]. The pathological hallmark of PD is the accumulation of insoluble inclusions consisting predominantly of synuclein, (Lewy bodies) mainly in the nigral dopaminergic neurons [61].

The study of toxin-induced parkinsonian syndromes has helped in elucidating PD pathogenesis. In the 20th century, exposure to 1-methyl-phenyl 4-phenyl-1,2,3,6-tetrahydropyridine was shown to cause symptoms resembling severe parkinsonism [319] by interfering with complex I of the ETC. Further, in experimental setting, rotenone, a complex I inhibitor, led to apoptosis in human neuroblastoma cells, and this finding was further expanded to discuss the involvement of pesticide exposure in the etiology of PD [320]. Subsequently, several researchers consistently reported a defect of complex I of the mitochondrial ETC leading to a 30–40% decline in its activity [48,321], caused by a diminished rate of production of complex I subunits, destruction of its structure, and oxidative damage [322]. α-synuclein targets to mitochondria and leads to a decrease in complex I activity [323]. LLRK2 (leucine-rich repeat kinase 2) mutations can increase α-synuclein levels [324]. PINK1 and Parkin regulate mitophagy, as described earlier. DJ1 is important for mitochondrial integrity and dynamics; overexpression of DJ1 has been shown to reduce mitochondrial fragmentation induced by rotenone, independently of PINK1 [325].

Convincing evidence has shown accumulation of increased lipid, protein, and DNA oxidation products in the degenerating neurons in PD [326,327,328] together with reduction in the antioxidant GSH [329], thereby implicating oxidative stress in the pathogenesis of PD. Initially, ROS probably originate from the ETC, external factors, and dopamine auto-oxidation [330], potentiated later by monoamine oxidase B metabolism of dopamine, inflammatory responses, or the contribution of heavy metals [331]. ROS have been shown to induce unorthodox activation of Iron Regulatory protein 1, thereby contributing to iron accumulation in dopaminergic cells [332]. Hydrogen peroxide can easily diffuse to adjacent neurons, where it can generate hydroxyl radicals through its interaction with iron [55]. PINK1 mutations in the kinase domain of the mitochondrially located molecule also increase the cells’ susceptibility to oxidative stress [55,333].

Another commonly described aspect in PD is impaired mitochondrial dynamics and altered mitochondrial morphology, with either elongated or fragmented mitochondria [334,335]. Impaired mitochondrial fusion, possibly related to the diminished levels of the short form of Opa1 described in brain samples of patients with idiopathic PD [336], contributes to the degeneration of nigral dopaminergic neurons, as does interaction of mutant LRRK2 with Drp1, which promotes mitochondrial fission [337]. α-synuclein can also bind to the OMM and decrease the mitochondrial fusion rate [338], which, together with increasing the Mfn1 and Mfn2 levels, promotes fragmentation and shortening of mitochondria [339]. In addition, mutant LRRK2 affects mitochondrial trafficking by interfering with MIRO1 removal, resulting in accumulation of MIRO1, which bind dynein and kinesin (microtubule motors) to mitochondria [340]. An interesting finding is the need to acidify the synaptic vesicles for proper loading of dopamine into these vesicles [341], a task performed by vacuolar ATPases and which require proper levels of ATP [330]. As such, in energy-deficient conditions, dopamine is improperly packed into synaptic vesicles and its cytoplasmic levels increase, creating conditions for its auto-oxidation [342].

### 6.3. Oxidative Stress in Amyotrophic Lateral Sclerosis

ALS is the third most common neurodegenerative disease, after AD and PD [16], occurring both sporadically (90–95% of cases) and as inherited disease caused by genetic mutations. Many mutations leading to familial ALS cases have been described, the most common ones being mutations in SOD1, FUS (fused in sarcoma/translocated in liposarcoma or heterogenous nuclear ribonucleoprotein P2), C9orf72 (chromosome 9 open reading frame 72), and TARDP (transactive response DNA binding protein 43) [343,344], the study of which can help identify the pathophysiologic mechanisms of the disease.

Decreased glucose metabolism with reduced ATP generation was reported in the cerebral cortex of SOD1^G93A^ mice before the clinical picture of ALS emerged, while the spinal cord exhibited these abnormalities only in later stages [345]. Similar abnormalities were observed in human patients as well [346], possibly related to downregulation of two key enzymes in glycolysis, PGK (phosphoglycerate kinase) and PGM2L1 (phosphoglucomutase-2-like 1) [347]. It appears that this metabolic disturbance does not affect neighboring astrocytes [348]. In response, neurons upregulate glycolysis [349] at the expense of increasing oxidative stress or may turn toward alternative sources of energy such as ketone bodies [350]. Beta-hydroxybutyrate facilitates oxidation of NADH and increases the NAD^+^/NADH ratio, thereby inhibiting mitochondrial ROS production [351] and activating SIRT1 and SIRT3 [31]. SIRT1, through deacetylation, alters the activity of the PGC-1α (peroxisome proliferator-activated receptor gamma coactivator 1-α)/ERR-α (estrogen-related receptor α) complex, with important roles in mitochondrial biogenesis regulation [350,352].

Disturbed cellular calcium handling is another feature described in motor neurons of patients with ALS as well as in vitro and in vivo models expressing mutant SOD1 [353]. After AMPA activation, the recovery of physiological calcium concentrations is delayed in motor neurons of ALS models [354], rendering them susceptible to Ca^2+^-induced excitotoxicity [355]. In addition, analysis of motor neurons from patients with TDP-43 mutations showed an upregulation of AMPA and NMDA receptors and an imbalance between MICU1 and MICU2 leading to reduced mitochondrial calcium uptake [356].

Mitochondrial quality control was also found deficient, with abnormally swollen and vacuolated organelles distributed mainly in the cell body and proximal axon of motor neurons [357]. Normally, mitochondrial mitophagic receptors OPTN and NDP52 target to mitochondria through their ubiquitin-binding domain and recruit autophagosomes [358]. Mutations of OPTN and TBK1, encoding an OPTN-containing kinase, were found in patients with ALS [359], leading to impairments of mitophagy and accumulation of damaged mitochondria which further decrease glucose metabolism and ATP generation. Excessive ROS activate Drp1, with consequent increased mitochondrial fission, while reduced ATP levels cause impaired autophagy and decrease proteasomal protein degradation with subsequent accumulation of protein aggregates which can trigger ER stress [360].

Although oxidative stress may not be the triggering factor, it is likely that it exacerbates ALS progression, and, again, mitochondria significantly contribute to the generation of ROS. The SOD1 mutation diminishes the affinity of Cu/ZnSOD for Zn, altering its antioxidant properties [361], and to a higher load of DNA damage, possibly due to a loss of nuclear protection [350]. The oxidants/antioxidants ratio is supplementally altered by the reduced levels of GSH found in the motor cortex of ALS patients in comparison with healthy volunteers [362]. The altered calcium homeostasis leads to mitochondrial dysfunction and to the activation of intrinsic (through mitochondrial release of death signals) or extrinsic (through specific ligands binding to death receptors like Fas or DR6—death receptor 6) pathways of apoptosis [363]. Motor neurons in affected regions of ALS patients show increased levels of p53 [364], possibly caused by reduced proteasomal degradation of p53 [365], which leads to a decrease in Bcl-2 and increase in Bax, Fas, and caspases 8 and 3 [366]. In addition, astrocytes and microglia are activated, leading to chronic inflammation and supplemental generation of ROS. Cultures of rat astroglia exposed to CSF from ALS patients showed increased release of inflammatory cytokines IL-6 and TNFα, reduced anti-inflammatory cytokine IL10, increased generation of cyclooxygenase-2 and prostaglandin E2, and downregulation of trophic factors such as GDNF (glial cell-derived neurotrophic factor) or VEGF (vascular endothelial growth factor) [367]. Moreover, astrocytes from ALS patients release neurotoxic factors able to damage motor neurons through a Bax-dependent mechanism [368] and proliferate to surround degenerating motor neurons and produce molecules which inhibit axonal regrowth [369]. Activated microglia are also toxic for motor neurons. In studying the effects of TDP43 mutation, microglial activation led to a NF-κB and NLRP3 (NLR family pyrin domain containing 3) inflammasome-dependent cascade which damaged motor neurons, while in the absence of microglia, motor neurons were able to survive [370]. Further, blocking the NF-κB signaling pathway in microglia rescued motor neurons in an animal model of ALS [362].

Table 2 summarizes the abnormal proteins associated with the three neurodegenerative diseases discussed and their deleterious actions on mitochondrial function.

## 7. Translating Theoretical Knowledge into Therapy

### 7.1. Targeting Oxidative Stress and Mitochondrial Dysfunction in Alzheimer’s Disease

Clinical trials on disease-modifying therapies have mostly failed, AD treatment proving one of the most challenging areas in modern medicine despite the increasing incidence and prevalence of the disease [387]. Currently, there are six FDA-approved drugs used for treatment of AD, four of which (tacrine, donepezil, rivastigmine, and galantamine) are acetyl cholinesterase inhibitors, memantine is a NMDA antagonist, while the very controversially recently approved aducanumab is an amyloid beta-directed monoclonal antibody [388]. Except for the last molecule, none of them are disease-modifying.

In a long list of trials marked by inconclusive results or failures, several antioxidant molecules have been repeatedly evaluated.

Curcumin is able to scavenge free radicals (ROS or reactive nitrogen species) [389], inhibit the activity of enzymes involved in free radical generation such as cyclooxygenases and xanthine oxidase [390], to modulate the activity of endogenous antioxidants such as glutathione peroxidase, catalase, and superoxide dismutases [391], to diminish lipid peroxidation, and to reduce the expression of NF-κB, ERK, inducible nitric oxide synthase, and inflammatory cytokines IL-1β and IL-6 [392]. The efficacy of 24-week curcumin supplementation on AD progression has been evaluated with inconclusive results in a phase 2 randomized, double-blind, placebo-controlled trial (NCT 00099710) [393], followed by a second pilot study of curcumin and Gingko extracts administered over 6 months (NCT00164749), the results of which were not released to date [394]. Another active trial, NCT01811381, aims at evaluating the efficacy of curcumin in association with yoga in patients with mild cognitive impairment [394].

Resveratrol has been shown to enhance the PI3K/Akt pathway and the nuclear translocation of Nrf2 [395], to suppress NF-κB and MAPK pathway activation, and to attenuate the microglial release of TNF-α and pro-inflammatory IL-1β [396]. A phase 2, double-blind, placebo-controlled study (NCT01504854) of the effect of resveratrol supplements in patients with probable AD has not yet published the results [394]. NCT00678431 is another completed study evaluating whether dietary supplementation with Resveratrol, glucose, and malate is able to slow the progression of AD. According to the published results, the differences between the active and placebo arms were not statistically significant [397]. A currently recruiting phase 1 study (NCT02502253) will assess BBB penetration and bioavailability of a grape seed extract as well as its efficacy in mild cognitive impairment, prediabetes, and type 2 diabetes mellitus [394].

As an antioxidant, quercetin scavenges free radicals [398], upregulates antioxidant enzymes such as glutathione transferase, glutathione peroxidase, SOD, catalase and thioredoxin [398], and induces the Nrf2-ARE pathway [399]. The SToMP-AD trial (Senolytic Therapy to Modulate Progression of Alzheimer’s Disease, NCT04063124) is an ongoing phase I/II trial assessing bioavailability and safety of dasatinib + quercetin in old adults with early-stage AD, while ALSENLITE, NCT04785300 is currently recruiting by invitation participants in a phase I/II trial to assess safety and tolerance of the combination [394].

Sulforaphane was able in animal models of AD to modulate the Nrf2/ARE pathway, to inhibit NFκB and upregulate neurotrophin expression [400]. An ongoing randomized, double-blind, placebo-controlled trial (NCT04213391) aims at assessing safety and efficacy of sulforaphane in mild to moderate Alzheimer’s disease patients [394].

Modest cognitive beneficial effects in AD patients were obtained with another dietary antioxidant supplement, soy isoflavone, in a placebo-controlled, double-blind pilot study (NCT00205179) which randomized 73 patients [401]. 8-hydroxydaidzein, a compound from fermented soy, exhibits antioxidant properties by quenching ROS, inhibiting microglial TNF-α and IL-6 release, and by upregulating the Nrf2 antioxidant and Akt/NF-κB anti-inflammatory pathways [402].

Chlorogenic acid, the main compound of coffee, was able in vitro to reduce intracellular ROS accumulation, stop the activation of α-secretases, BACE-1, or MAPK, and attenuate GSH depletion [403]. In animal models of AD, polyphenols from coffee reduced hippocampal amyloid plaque burden, thereby attenuating memory impairments and cognitive dysfunction [404]. CAFCA (NCT04570085) is a multicenter, randomized, double-blind, placebo-controlled trial planned by the University of Lille, aiming at evaluating the effect of a 30-week caffeine treatment on cognitive function in early and moderate stages of Alzheimer’s disease [394].

Lipoic acid is a molecule with anti-inflammatory functions, able to reduce NF-κB activity in vitro in cells stimulated with TNF-α [405], and able to recycle vitamins C, E, and glutathione [406]. A small phase I/II trial randomized 39 AD patients to receive lipoic acid, fish oil, or placebo and evaluated the rate of cognitive impairment over 12 months, showing promising results [407] and has been followed by another phase I/II study, NCT01058941, with 67 participants followed over 18 months, the results of which have not yet been published.

Other trials with antioxidants have also been performed or are under way. For example, NCT00117403 was a phase 1 trial with the active arm taking a combination of vitamins E, C, lipoic acid, and coenzyme Q10 for 4 months. Although CSF analysis indicated a reduction of markers of oxidative stress, the investigators cautioned against a more rapid cognitive decline [408]. A prospective cohort study with 4246 participants (PREADVISE, NCT00040378) followed for 7–12 years concluded that neither vitamin E nor selenium could influence the rate of cognitive decline [409]. Moreover, a phase 3 trial comparing the added effect of tocopherol (TEAM-AD, NCT00235716) revealed that vitamin E showed a modest benefit over placebo in slowing cognitive decline but did not add to the effect of memantine [410]. A currently recruiting phase 1 study, NCT04430517, will assess the effect of oral 1000 mg of nicotinamide riboside intake for 12 weeks on redox status, GSH levels, and mitochondrial function as well as on cognition in participants with mild cognitive impairment od AD [394], while a phase 1 trial of supplementation of glycine, alanine, and N-acetylcysteine (Glutathione in Alzheimer’s disease, NCT04430517) for 24 weeks is registered but not yet recruiting [394].

Latrepirdine has been shown in preclinical studies to prevent lipid peroxidation and inhibit opening of the MPTP as well as voltage-gated calcium ion (Ca^2+^) channels in neurons, protecting against Aβ-induced neurotoxicity [411], while having very low acetylcholinesterase-inhibitory action, as opposed to other antihistamine drugs. In an 8-week open-label pilot study on 14 AD patients, latrepirdine showed clinical benefits [411], which prompted a phase 2 randomized, double-blind, placebo-controlled trial of 60 mg latrepirdine orally for 26 weeks in 183 AD patients (NCT00377715), which confirmed the positive effect on cognition, behavior, and global function [412]. However, the CONNECTION trial (NCT00675623), a phase 3 clinical trial of 60 mg latrepirdine for 6 months in 598 patients could not demonstrate any significant effect of the drug compared to placebo [413], and neither could CONCERT (NCT00829374), a phase 3 clinical trial comparing two doses of latrepirdine to placebo in 1003 patients. A meta-analysis of the several studies with latrepirdine in mild-to-moderate AD patients concluded that despite some modest beneficial effects on behavior, cognition and functional status are not influenced [414].

### 7.2. Targeting Oxidative Stress and Mitochondrial Dysfunction in Parkinson’s Disease

Currently prescribed treatments for PD are levodopa with 1-amino acid decarboxylase inhibitors, dopamine agonists (pramipexole, ropinirole, rotigotine), MAO-B inhibitors (selegiline, rasagiline), catechol-O-methyltransferase inhibitors (entacapone), and anticholinergic drugs [415], while refractory cases may undergo surgical deep brain stimulation procedures. Although helpful in relieving symptoms, none of these approaches interfere with the neurodegenerative process, and some concerns regarding the accelerated neurodegeneration caused by levodopa metabolism and subsequent increase in oxidative stress have been raised [416]. Recent developments in the treatment of PD focus on [417] immunotherapies to restrict the propagation of α-synuclein, neurotrophic factors, such as GDNF (glial cell-derived neurotrophic factor), regenerative therapies using cell-based and genetic approaches to replace the function of the lost dopaminergic neurons, re-establishing the balance between neurotransmitters by targeting non-dopaminergic neurotransmission, interfering with the neuroinflammatory response, or improving the stereotactical surgical treatments. As for supporting/improving mitochondrial function and targeting oxidative stress, the clinical trials performed so far and their conclusions are described below.

Creatine acts as an antioxidant and improves mitochondrial function [418]. Although a pilot study reported in 2006 could not detect any improvement in the UPDRS scores of PD patients after 2–4 g creatine/day for 2 years [419], a subsequent phase 2 multi-center, double-blind, pilot study of minocycline and creatine was conducted in 195 patients with early untreated PD to test efficacy in slowing disease progression (National Institute for Neurological Disorders and Stroke Parkinson’s Disease Neuroprotection Trial, NCT00063193) and showed promising results [420], followed by a larger phase 3 double-blind, parallel-group, placebo controlled study (NET-PD LS-1, NCT00449865), which enrolled 1741 participants who were administered 10 creatine/day for 5 years, which failed to show improvements in clinical outcome [421].

Vitamin E has also been evaluated in several studies, with mixed results. After a population-based study showed a negative association between vitamin E intake and incident PD [422], similar subsequent studies failed to confirm the decreased PD risk associated with dietary antioxidants [423]. A pilot open label study evaluated the efficacy of tocopherol in slowing down the disease progression and claimed that vitamin E can postpone the need for levodopa therapy by 2.5 years [424], a claim contradicted by the findings of a subsequent multicenter, randomized, placebo-controlled study (DATATOP) carried out on 800 patients to evaluate the effect of selegiline and/or tocopherol versus placebo on the onset of disability prompting the need for levodopa therapy [425] and which found beneficial effects for selegiline but not for tocopherol. At present, a phase 2 pilot, randomized, double blind, placebo-controlled trial (NCT04491383) is recruiting 100 participants in Singapore to assess efficacy of tocotrienols delaying motor disability in PD [394].

Since complex I activity was found reduced in PD, coenzyme Q10, an antioxidant and electron acceptor for complexes I and II seemed a reasonable approach. A randomized, double-blind, placebo-controlled trial which enrolled 80 early-stage PD patients showed that 300–1200 mg coenzyme Q10/day could significantly reduce disability in a dose-dependent manner [426]. However, a large phase 3 multi-center, randomized, double-blind, placebo-controlled clinical trial which included 600 participants (QE3, NCT00740714) failed to show clinical benefits for 1200 or 2400 mg of coenzyme Q10 daily [427]. To overcome the reduced BBB permeability of coenzyme Q10, it was delivered in a nanodispersed solution in doses of 300 mg (equivalent to 1200 mg coenzyme Q10)/day to 132 participants in a phase 3 clinical trial (NCT00180037), which also showed null results [428]. A mitochondria-targeted synthetic coenzyme Q10 analog, MitoQ, has positive charges and lipophilic properties, enabling it to easily cross the BBB and accumulate within mitochondria. However, it also failed to alter the disease course in a phase 2 clinical trial which enrolled 128 participants (NCT00329056) [429].

Glutathione is an endogenous antioxidant molecule which has been tested in a number of clinical trials. While the intravenous administration of 1200 mg/day of reduced glutathione was shown to significantly improve disability [430], a subsequent trial with 700 mg glutathione/day given also IV failed to confirm these results [431]. After a phase 1 safety trial (NCT01398748), intranasally administered glutathione was tested in a phase 2b study on 45 participants over 12 weeks for efficacy on disease progression ((in) GSH, NCT02424708) but showed null results [432]. Studies with other dietary antioxidants are in preclinical phases, but, considering the poor BBB penetration of these compounds [433], it is unlikely that they will successfully translate in clinical settings.

Melatonin, being amphiphilic, can cross the BBB and exhibit antioxidant activity in the central nervous system. After promising results in animal models, the effect of 3 mg melatonin/day for 4 weeks on motor performances and quality of sleep was assessed in a small study involving 18 PD patients. Sleep disturbances are common complaints in PD and may herald a more aggressive course and a more rapid progression toward cognitive decline [434]. The trial showed that melatonin improved the subjective quality of sleep but had no effect on motor performances [435].

Given the high levels of iron in nigral neurons and the involvement of iron in ROS production, the efficacy of iron chelators was assessed first in a pilot study on 22 participants (DeferipronPD, NCT01539837) and showed non-significant motor improvement [436] followed by a phase 2/3 trial enrolling 37 patients in the active arm and 40 in the placebo arm (FAIR PARK-I, NCT00943748) and which showed reduction of iron in the substantia nigra as well as motor improvement [437]. Currently, an extended clinical trial, enrolling 372 participants (FAIR PARK-II, NCT02655315), is active but not recruiting [394].

Other agents, such as PPARγ coactivator-1α (PGC-1α) agonists, Nrf2 enhancers, or natural antioxidant compounds are only in preclinical stages [415] except for pioglitazone, a PPARγ coactivator-1α agonist, which, administered in doses of 15–45 mg/day in 210 PD patients (NCT01280123) could not show significant motor benefits [438].

### 7.3. Targeting Oxidative Stress and Mitochondria in ALS

For years the only approved therapy in ALS was riluzole, a glutamate antagonist which interferes with excitotoxic neuronal death [439].

Based on the results of several studies evaluating the effect of edaravone (3-methyl-1-phenyl-2-pyrazoline-5-one, or MCI-186), the FDA approved the molecule in 2017 for use in ALS [440]. Edaravone acts as a free radical scavenger of hydroxyl and peroxyl radicals, hydrogen peroxide and peroxynitrite [441], and activates the Nrf2/HO-1 signaling pathway, protecting cells against apoptosis [440], thereby offering a modest clinical benefit.

Melatonin, an endogenous molecule involved in regulation of the sleep–wake cycle, exhibits also antioxidant properties [442]. In ALS patients it slowed the progression of the motor impairment [443]. However, no clinical trial with melatonin in ALS is currently ongoing [394].

Alpha-lipoic acid is a hydroxyl radical scavenger and induces the ERK/PI3K/Akt pathway, thereby regulating the expression of antioxidant genes. Its safety and efficacy in ALS are currently being explored and compared to riluzole in the Explore Neuroprotective Effect of Lipoic Acid in Amyotrophic Lateral Sclerosis (NCT04518540) trial, conducted by the Zhejiang University School of Medicine [394].

Dopaminergic drugs, such as pramipexole, reduce oxidative stress [444] and glutamate excitotoxicity [445]. The safety and efficacy of dexpramipexole was evaluated in several phase 1, 2, and 3 clinical studies. So far, results published from a phase 3 randomized, double-blind, placebo-controlled, multi-center study which enrolled 942 participants (EMPOWER, NCT 01281189) revealed a good safety profile but non-significant efficacy of the treatment arm compared to placebo [446] despite promising results of a phase 2 randomized, double blind safety and tolerability study (NCT00647296) [447]. The results of the extension phase, NCT01622088 are still awaited [394]. Another drug currently used in the treatment of PD, rasagiline, a monoamine oxidase B inhibitor, exhibits also antioxidative and anti-apoptotic activities, making it a potential therapeutic option in ALS. Its safety and efficacy were evaluated in two phase 2 trials, an open-label one enrolling 36 participants (NCT01232738), which showed no improved clinical course but suggested reduced apoptosis [448] and a second phase 2, double-blind, placebo-controlled trial (NCT017866030) evaluating efficacy, which showed null results after 12 months of treatment [449].

Dietary antioxidants and antioxidant food supplements have also been tested in several trials. A phase 2 trial with high doses of coenzyme Q10 in ALS (NCT00243932) concluded that there is insufficient evidence to justify a phase 3 trial [450], while NCT02588807, a phase 1 trial with food supplements for the treatment of patients with ALS is suspended [394]. However, two further studies, planned but not yet recruiting, will evaluate safety and efficacy of coenzyme Q10 with vitamin E, N-acetyl cysteine, and L-cysteine (MICABO-ALS, NCT04244630) and of liposomed polyphenols resveratrol and curcumin (NCT04654689) in patients with ALS.

## 8. Concluding Remarks

From the repeated failures of drugs targeting mitochondrial dysfunction and oxidative stress in neurodegenerative diseases, it appears that starting these interventions by the time of clinical diagnosis is probably too late. Due to the resilience of the brain to insults, the described pathogenetic cascades and loops are already full-blown and significant neuronal loss has already occurred when clinical symptoms enable diagnosis. Genetic testing in familial neurodegenerative diseases could allow starting these therapies in the preclinical stage. Since the present healthcare systems cannot afford extensive and invasive evaluations of the population at large, promoting a healthy lifestyle, with plenty of dietary antioxidant intake and avoidance of exogenous oxidants could postpone the onset of neurodegenerative diseases. Hopefully, ongoing research will provide more efficient, multimodal molecules to interfere with the pathogenesis of these diseases, which, due to aging of the population, are a global threat.

## Figures and Tables

**Table 1:** The modified mitochondrial proteins described in AD and their involvement in cellular processes (adapted from Jové et al. [291]).

Protein	Biological Process	Reference
Glutamate dehydrogenase 1	TCA cycle (energy metabolism)	[294,295]
Malate dehydrogenase	TCA cycle (energy metabolism)	[295,296]
Subunit 5a of cytochrome c oxidase	ETC (energy metabolism)	[297]
NADH dehydrogenase (ubiquinone)	ETC (energy metabolism)	[297]
Subunits alpha, beta, d, and o of ATP synthase	OXPHOS (energy metabolism)	[291,297,298]
Core protein 1 of ubiquinol-cytochrome c reductase complex	ETC (energy metabolism)	[298]
Glutamine synthetase	Neurotransmission	[297,298]
Manganese superoxide dismutase	Antioxidant	[290,295]
Protein 1 of voltage-dependent anion-selective channels	Ion channel	[297]

TCA—tricarboxylic acid cycle; ETC—electron transport chain; OXPHOS—oxidative phosphorylation.

**Table 2:** Abnormal proteins associated with impaired mitochondrial function in the three most frequent neurodegenerative diseases (adapted form Paß et al. [72]). ↑ = increases; ↓ = decreases.

Protein	Associated Disease	Result of Malfunction	References
**Mitochondrial Fission**			
Aβ	AD	↑ Drp1 levels	[371]
LRRK2	PD	↑ mitochondrial fission	[372]
SOD-1, TDP-43	ALS	↑ Drp1 and FIS1 protein levels (increases mitochondrial fission)	[373]
VPS35	PD	Increased turnover of Drp1 and increased mitochondrial fission	[374]
**Mitochondrial Fusion**			
Aβ	AD	↑ Mfn1, Mfn2, OPA1 protein levels	[371]
α-synuclein	PD	↓ mitochondrial fusion	[338]
LRRK2	PD	↓ OPA1 protein levels	[335]
SOD-1, TDP-43	ALS	↓ Mfn1 and OPA1 protein levels (decreased mitochondrial fusion)	[373,375]
VPS35	PD	↓ Mfn2 protein levels (decreased mitochondrial fusion)	[376]
**Mitochondrial Transport**			
Aβ	AD	↓ mitochondrial transport	[303]
LRRK2	PD	Accumulation of MIRO1 (decreased mitochondrial transport)	[377]
Tau	AD	↓ axonal transport	[378]
**Mitochondrial Degradation**			
Aβ	AD	Delayed removal of damaged mitochondria	[379]
LRRK2	PD	Delayed removal of damaged mitochondria	[377]
OPTN	ALS	Delayed removal and accumulation of damaged mitochondria	[380,381]
p62	ALS	Impaired LC3 recognition Decreased autophagy	[382]
Parkin	PD	Impaired mitophagy in neurons and axons Degeneration of nigral dopaminergic neurons with impaired mtDNA replication	[383,384]
PINK1	PD	Decrease of mitochondrial membrane potential	[385]
VPS35	PD	Delayed removal of damaged mitochondria	[386]

AD—Alzheimer’s disease; ALS—amyotrophic lateral sclerosis; PD—Parkinson’s disease.
